# Progression and life expectancy in primary lateral sclerosis

**DOI:** 10.1136/jnnp-2025-336037

**Published:** 2025-05-16

**Authors:** David G Lester, Alexander G Thompson, Kevin Talbot, Martin R Turner

**Affiliations:** 1 Nuffield Department of Clinical Neurosciences, University of Oxford, Oxford, UK

**Keywords:** MOTOR NEURON DISEASE, NEUROEPIDEMIOLOGY, Prognosis, STATISTICS, NEUROMUSCULAR

## Abstract

**Objectives:**

To characterise the clinical characteristics and longitudinal outcomes in primary lateral sclerosis (PLS), including median survival from symptom onset and age at death.

**Methods:**

The authors retrospectively reviewed electronic health records of patients diagnosed with PLS referred to a specialised motor neuron disorders clinic from 2002 to 2024, analysed longitudinal Revised Amyotrophic Lateral Sclerosis Functional Rating Scale (ALSFRS-R) assessments using joint models and used Kaplan-Meier methods and life tables to calculate median survival and age at death compared with population-based values.

**Results:**

Of 52 patients, 34 (65%) were male, 41 (79%) first noted symptoms in the lower limbs and 10 (19%) in corticobulbar function. Median age of symptom onset was 53 years. The mean annual rate of functional decline was −1.92 ALSFRS-R points (95% CI −3.03 to −0.78), with equal highest rates of decline in fine and gross motor subscores. Five patients (10%) received gastrostomy and three (6%) non-invasive ventilation. Median survival from symptom onset was 23.1 years (22.7 to not reached), and median age at death was 79.5 years (77.8 to not reached) compared with a population-based reference mean of 81.9 years (81.1 to 82.8).

**Discussion:**

PLS may be commensurate with near-normal life expectancy. Significant disability arises from limb motor dysfunction, with a minority of patients requiring nutritional or respiratory support. This has important implications for counselling and trial design.

## Introduction

 First described in the 19th century by Charcot and Erb, primary lateral sclerosis (PLS) is a rare motor neuron disorder of unknown aetiology, characterised by slowly progressive clinical upper motor neuron (UMN) dysfunction.^1 2^ Insidious muscle stiffness impairs balance and mobility, particularly in the lower limbs, typically followed by corticobulbar involvement. According to consensus criteria in 2019 diagnosis remains clinical and requires a minimum age of 25 years, progressive UMN symptoms for at least 2 years, UMN signs in at least two of three body regions (lower limbs, upper limbs and bulbar), the absence of unexplained sensory symptoms, the absence of active lower motor neuron degeneration and the absence of an alternative diagnosis demonstrated by neuroimaging or biofluid analysis.^3^ The mainstays of management are symptomatic relief of spasticity, emotional reflex hypersensitivity, sialorrhoea and dysarthria, ideally delivered through an interdisciplinary team.^4^ Approximately 7% of cases have been associated with presumed pathogenic variants found within screening panels for a broader range of neurodegenerative disorders, but with poorly understood phenotypic specificity and penetrance.^5^ PLS is generally considered to be part of the spectrum of amyotrophic lateral sclerosis (ALS), although an extreme, with limited studies demonstrating the hallmark pathology of ALS, namely neuronal and glial cytoplasmic inclusions of aggregated phosphorylated transactive response DNA binding protein of 43 kDa (TDP-43).^6^

Due to slow disease progression and dropout during longitudinal analysis, there are no established estimates for median disease duration or age at death calculated using Kaplan-Meier methods in PLS. Instead, mean or unadjusted disease duration approximations ranging from 9 to 19 years have been published.^57^,^11^ Similarly, little is known about the natural history of PLS as measured by a functional rating scale. In 2020, an unadjusted mean annual rate of functional decline measured by the Revised Amyotrophic Lateral Sclerosis Functional Rating Scale (ALSFRS-R) total score (48–0) of −1.6 points in 195 PLS patients over a median follow-up time of 3 years was published by the multinational registry Northeast ALS Consortium (NEALS).^7^ A more complete understanding of these long-term outcomes in PLS would enrich patient counselling and clinical decision-making, and potentially guide power calculations for detection of rate of change in disease-modifying treatment trials.

## Methods

We reviewed the electronic health records of all patients diagnosed with PLS from 2002 to 2024 by experienced neurologists at the Oxford Motor Neuron Disease Care and Research Centre. Date of birth, sex, date and site of symptom onset, clinical genetic test results, date and score of historic ALSFRS-R assessments, date of gastrostomy and non-invasive ventilation (NIV), and date of death or censorship were ascertained. Clinical genetic testing was performed using routinely available whole genome sequencing on a panel of 123 genes most commonly associated with adult-onset neurodegenerative disorders.^12^ Patients were contacted by telephone for an updated ALSFRS-R assessment and censoring time.

Joint models with fixed effects for time, a random slope and intercept for each patient, and a Cox proportional hazards submodel with no additional covariates were used to estimate the longitudinal change in total ALSFRS-R and its subscores. For each patient, a life expectancy value specific to the year of symptom onset was obtained from the Office for National Statistics’ life tables for England, matched by sex and age at symptom onset.^13^ Kaplan-Meier estimators were used to calculate median time from symptom onset to death and median age at death in PLS, and log-rank tests were used to compare the survival distributions of sex and onset site in PLS. Statistical analysis was performed using R (V.4.4.0).

## Results

### Demographics and presenting characteristics

Of 69 patients initially identified as having been diagnosed with PLS, 17 (25%) were excluded from further analysis because of unavailable primary documentation (n=3, 4%) or diagnostic revision to ALS (n=14, 20%). Of the remaining 52 patients, 34 (66%) were male, and the median age of symptom onset was 53 years (table 1). In 41 patients (79%), symptoms were first noticed in the lower limbs, 10 patients (19%) reported first symptoms in the corticobulbar territory, and one patient presented with a progressive spastic hemiparesis consistent with the clinical phenotype described by Mills.^14^ No patients presented with isolated upper limb symptoms. Clinical genetic testing was conducted on five patients: four had negative results, and one showed a heterozygous c.1747G>A variant in exon 11 of the microtubule-associated protein tau (*MAPT*) gene. In 2017, this variant was predicted by the clinical genetics service provider to cause a conservative valine to methionine substitution at position 583 in a functional protein domain and was classified as a variant of uncertain significance.

**Table 1:** Patient demographics

	Overall
	N=52
Sex	
Female	18 (35%)
Male	34 (65%)
Site of onset	
Bulbar	10 (19%)
Lower limb	41 (79%)
Hemiparesis	1 (2%)
Age at onset (years)	
Median (SD)	53 (9)
Pathogenic variant	
*MAPT* VUS (p.Val583Met)	1 (2%)
Negative	4 (8%)
Not tested	47 (90%)
Gastrostomy	
Yes	5 (10%)
No/unknown	47 (90%)
Non-invasive ventilation	
Yes	3 (6%)
No/unknown	49 (94%)

p.Val583Met=valine to methionine substitution at amino acid position 583.

MAPT, microtubule-associated protein tau gene; SD, Standard deviation; VUS, variant of uncertain significance.

### Clinical assessments and outcomes

**Figure 1:**
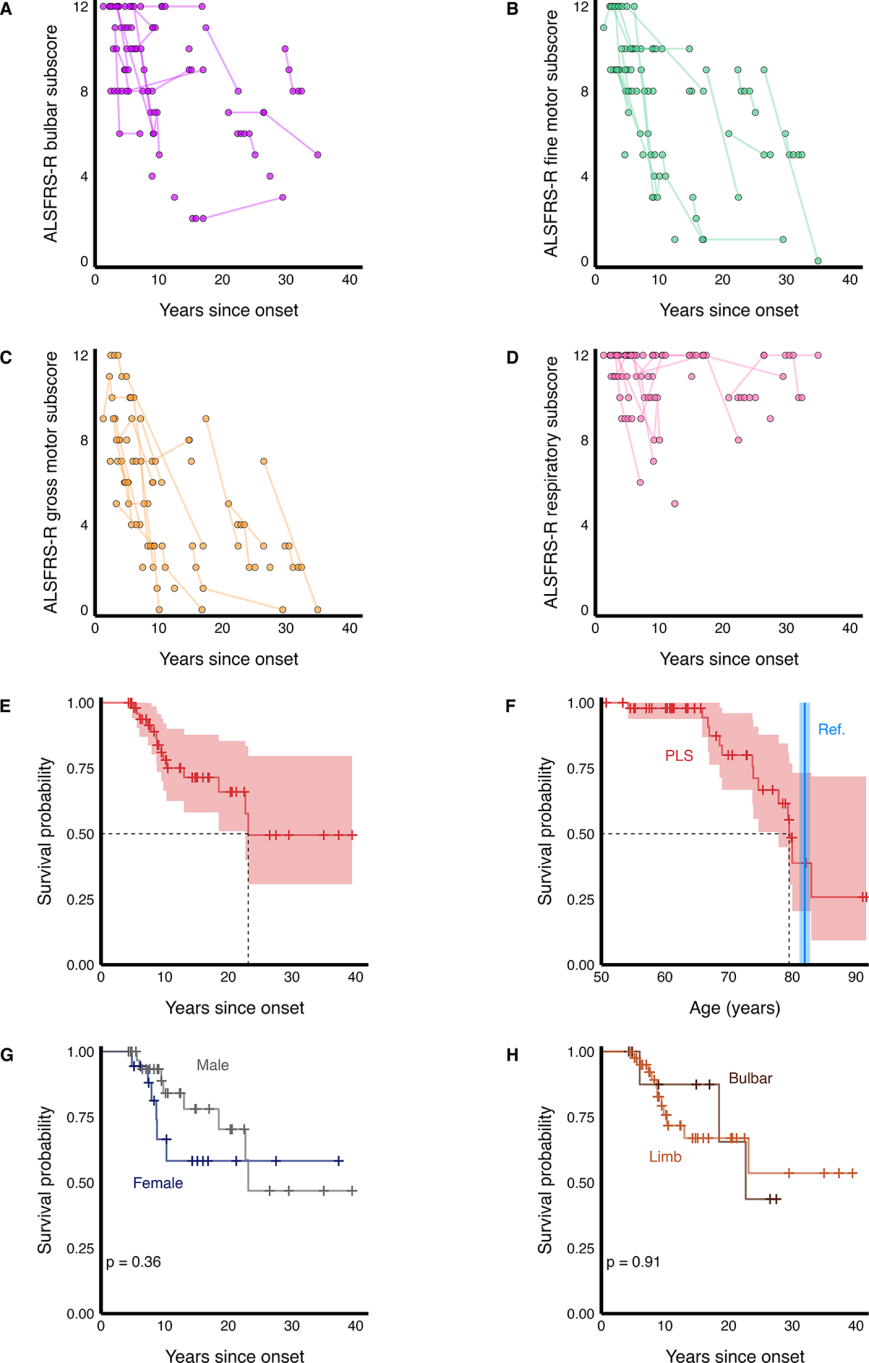
(A–D) ALSFRS-R bulbar, fine motor, gross motor and respiratory subscores, with longitudinal measurements within individuals connected by line. (E) Kaplan-Meier curve of cumulative survival probability following primary lateral sclerosis (PLS) disease onset, shown with 95% CIs. (F) Kaplan-Meier curve of cumulative survival probability for PLS patients by overall age versus population-based mean life expectancy, both shown with 95% CIs. (G) Kaplan-Meier curves of cumulative survival probabilities following disease onset stratified by sex, (H) and by onset site, both shown with log-rank p-values. ALSFRS-R, Revised Amyotrophic Lateral Sclerosis Functional Rating Scale; PLS, primary lateral sclerosis; Ref., reference.

In total, 81 ALSFRS-R assessments were performed on 33 patients (63%) (figure 1A–D). Of these, 18 were contemporaneous, while the remaining assessments were historic. Joint modelling determined mean annual rates of change in the ALSFRS-R bulbar, fine motor, gross motor and respiratory subscores of −0.33 (95% CI −0.58 to −0.08), –0.64 (−0.98 to −0.30), –0.64 (−0.94 to −0.36) and −0.22 (−0.50 to 0.06) points/year, respectively, with a mean annual rate of change in ALSFRS-R total score of −1.92 points (−3.03 to −0.78). Over a median follow-up time of 10 years, 14 patients died and 38 were censored. Causes of death were not reviewed. Median survival from disease onset was 23.1 years (22.7 to not reached), and median age at death was 79.5 years (77.8 to not reached) compared with a mean of 81.9 years (81.1 to 82.8) in the population-based matched life expectancies (figure 1E,F). There were no significant survival differences when stratified by sex (p=0.36) or onset site (p=0.91) (figure 1G,H). Five patients (10%) had gastrostomy inserted, after between 7 and 10 years’ disease duration, and three patients commenced NIV after between 6 and 10 years disease duration. Notably, the indication for two of the three patients who received NIV was obstructive sleep apnoea rather than primary neuromuscular dysfunction.

## Discussion

We report the clinical characteristics and longitudinal outcomes of a large single-centre cohort of PLS patients (n=52). Unlike the sex parity noted by NEALS, but similar to a large Dutch population-based study, we describe a male predisposition in PLS.^5 7^ At 53 years, the median age of onset in our cohort is in keeping with the published literature.^57^,^11^ Similarly, we report the lower limbs as the single most common site of symptom onset, with the rest being corticobulbar.^58^,^11^ We suggest the reported onset of symptoms solely in the upper limbs should be a ‘red flag’ for an alternative diagnosis.

The low gastrostomy and NIV rates in our cohort (10% and 6%) are close to those described in the NEALS cohort (7% and 3%).^7^ However, these rates may be underestimates as our analysis does not capture all patients in advanced disease states. The median disease duration of 23.1 years and median age at death of 79.5 years in this cohort were uniquely derived using Kaplan-Meier methods. Compared against a population-based reference mean survival of 81.9 years in this cohort, this suggests that PLS diagnosis is compatible with near-normal life expectancy, although with significant progressive physical disability and secondary mental distress. The longitudinal ALSFRS-R assessment analysis revealed joint highest rates of functional decline in fine motor and gross motor domains, with slower and slowest changes seen in bulbar and respiratory subscores, and an overall annual rate of change of −1.92 points.

The principal limitations of this study are the use of a single centre and retrospective design, which risk unrepresentative patient sampling and inconsistent data collection. A population-based analysis would provide more precise and confident survival estimates. Use of the more recently developed PLS Functional Rating Scale might have more sensitive assessments of disability compared with ALSFRS-R.^15^ The data do not integrate neurophysiology, neuroimaging or individual-level features, and the differentiation of PLS from UMN-predominant forms of ALS remains challenging and requires international effort, perhaps through the establishment of a global registry. This challenging differentiation should be acknowledged when discussing prognosis with those suspected to have PLS and bounds the applicability of the analysis presented in this work to those with a certain diagnosis. Few patients underwent genetic testing; systematic testing is needed, but no consistent monogenetic cause of PLS has been identified to date. The main strengths of this work are the long follow-up period that allowed for exclusion of those who were ultimately diagnosed with ALS and the use of survival analysis.
